# Self-recoverable elastico mechanoluminescence of a hybrid metal halide crystal

**DOI:** 10.1093/nsr/nwae372

**Published:** 2024-10-21

**Authors:** Tian-Yi Yang, Si-Nuo Li, Hai-Sheng Chen, Zi-Ying Li, Zhi-Gang Li, Rui Feng, Fei-Fei Gao, Ying Zhang, Yi-Ming Liu, Yang Zhang, Wei-Wei Liu, Wei Li, Xian-He Bu

**Affiliations:** School of Materials Science and Engineering & Tianjin Key Laboratory of Metal and Molecule-Based Material Chemistry, Nankai University, Tianjin 300350, China; School of Materials Science and Engineering & Tianjin Key Laboratory of Metal and Molecule-Based Material Chemistry, Nankai University, Tianjin 300350, China; Institute of Modern Optics & Tianjin Key Laboratory of Micro-Scale Optical Information Science and Technology, Nankai University, Tianjin 300350, China; School of Materials Science and Engineering & Tianjin Key Laboratory of Metal and Molecule-Based Material Chemistry, Nankai University, Tianjin 300350, China; School of Materials Science and Engineering & Tianjin Key Laboratory of Metal and Molecule-Based Material Chemistry, Nankai University, Tianjin 300350, China; School of Materials Science and Engineering & Tianjin Key Laboratory of Metal and Molecule-Based Material Chemistry, Nankai University, Tianjin 300350, China; School of Materials Science and Engineering & Tianjin Key Laboratory of Metal and Molecule-Based Material Chemistry, Nankai University, Tianjin 300350, China; School of Materials Science and Engineering & Tianjin Key Laboratory of Metal and Molecule-Based Material Chemistry, Nankai University, Tianjin 300350, China; School of Materials Science and Engineering & Tianjin Key Laboratory of Metal and Molecule-Based Material Chemistry, Nankai University, Tianjin 300350, China; Institute of Modern Optics & Tianjin Key Laboratory of Micro-Scale Optical Information Science and Technology, Nankai University, Tianjin 300350, China; Institute of Modern Optics & Tianjin Key Laboratory of Micro-Scale Optical Information Science and Technology, Nankai University, Tianjin 300350, China; School of Materials Science and Engineering & Tianjin Key Laboratory of Metal and Molecule-Based Material Chemistry, Nankai University, Tianjin 300350, China; School of Materials Science and Engineering & Tianjin Key Laboratory of Metal and Molecule-Based Material Chemistry, Nankai University, Tianjin 300350, China

**Keywords:** elastico mechanoluminescence, self-recovery, hybrid metal halide, piezoelectric, phase transition

## Abstract

Materials exhibiting self-recoverable elastico mechanoluminescence (EML) are highly sought after due to their utility in sensing and information encryption. However, the current pool of identified EML materials remains limited, exclusively comprising purely inorganic substances. This study delves into the investigation of the EML properties of a zero-dimensional (0D) organic-inorganic metal halide denoted as [C_19_H_18_P]_2_MnBr_4_ (where C_19_H_18_P^+^ signifies methyl triphenyl phosphonium). Notably, [C_19_H_18_P]_2_MnBr_4_ manifests two distinct polymorphs, with the piezoelectric and non-piezoelectric polymorphs exhibiting thermodynamic and kinetic stability, respectively. Despite both compounds showing bright greenish luminescence, only the piezoelectric phase exhibits desirable EML behavior. The EML in this context is distinguished by its high intensity, strong fatigue resistance and prompt self-recovery. The underlying mechanism of EML in the piezoelectric polymorph can be explained by the piezoelectric effect and the stress-induced energy band tilting. Calorimetric and piezoelectric experiments reveal the self-recoverable EML arises from the relaxation of the stress-induced kinetically stable non-piezoelectric to the thermodynamically favored piezoelectric phase. This work paves a new pathway in the exploration of self-recoverable EML materials in the realm of hybrid organic-inorganic crystals.

## Teaser

A zero-dimensional hybrid metal halide was discovered to show self-recoverable elastico mechanoluminescence. The property is attributed to the intrinsic piezoelectric effect and the stress-induced energy band tilting, and its self-recovery arises from the stress-induced phase transition of the kinetically stable parent phase to a thermodynamically favored polymorph.

## INTRODUCTION

Mechanoluminescence (ML) is the phenomenon wherein luminescence is emitted upon the mechanical stimulation of a solid material [1–3]. Two specific categories called Fracto ML (FML) and Elastico ML (EML) refer to the luminescence induced by fracture and elastic deformation, respectively [4]. FML is prevalent in nearly half of inorganic salts and organic molecules, including daily consumed cube sugar, and is frequently encountered in nature. Although EML occurrences are less frequent compared to FML, their significance lies in the capacity to offer reproducible ML, rendering them more valuable for real applications. Two prominent examples of EML materials are rare earth elements doped SrAl_2_O_4_ and transition metal ion doped ZnS, both reported in 1999 [5,6]. Notably, the former requires exposure to ultraviolet radiation following mechanical stress to facilitate ML recovery [5]. Recent studies have explored alternative approaches for ML recovery, incorporating external energy supplements such as heating or exposure to solvents [7–9]. The latter, which needs no additional energy input after mechanical stimulation, is referred to as self-recovery EML [10,11]. The demand for self-recovery EML materials spans diverse fields, encompassing applications in information security, sensing, and illumination, yet they are extremely rare [12–14]. Comprehending and delineating the mechanism of ML, particularly concerning recovery, poses considerable challenges. Contributing factors to this complexity include inherently brief time scales, simultaneous occurrence of various deformation modes, and the involvement of multiple variables such as friction on the contact surface, ambient gas effects, and others [15–18]. Therefore, alongside the pursuit of novel ML materials, there is a concurrent need for innovative approaches to facilitate a deeper understanding of the mechanisms underlying ML and the self-recovery EML.

Zero-dimensional (0D) hybrid organic-inorganic metal halides (OIMH) have gained significant attention in the field of ML due to their outstanding optical properties, cost-effectiveness and ease of synthesis [19–24]. On the one hand, the absence of a framework structure between the inorganic halide units in 0D OIMH makes them prone to structural and property alterations under stress stimulation [25,26]. On the other hand, the isolation of inorganic halides at specific sites provides significant advantages for studying the complex ML process. Notably, manganese-based materials, among them, exhibit substantial reserves, low toxicity, robust stability and environmental friendliness, rendering them ideal candidates for ML materials [27,28].

In this report, two centimeter-sized 0D OIMH crystals, specifically [C_19_H_18_P]_2_MnBr_4_ were synthesized using the solution diffusion method under ambient conditions. The crystal structures belong to the *P*2_1_ and *R*${\mathrm{\bar{3}}}$*c* space groups, denoted as polymorph **1** and **2**, respectively. Crystal **1** demonstrated exceptional optical performance, characterized by bright luminescence with a high quantum yield. Not only FML but EML which only needs two minutes for recovery was observed in **1**. The mechanism of ML can be elucidated using the piezoelectric field theory. Differential scanning calorimetry (DSC) and piezoelectric measurements provide insights, revealing that fatigue and self-recovery are attributed to the phase transition between **1** and **2**.

## RESULTS AND DISCUSSION

**Figure 1.**
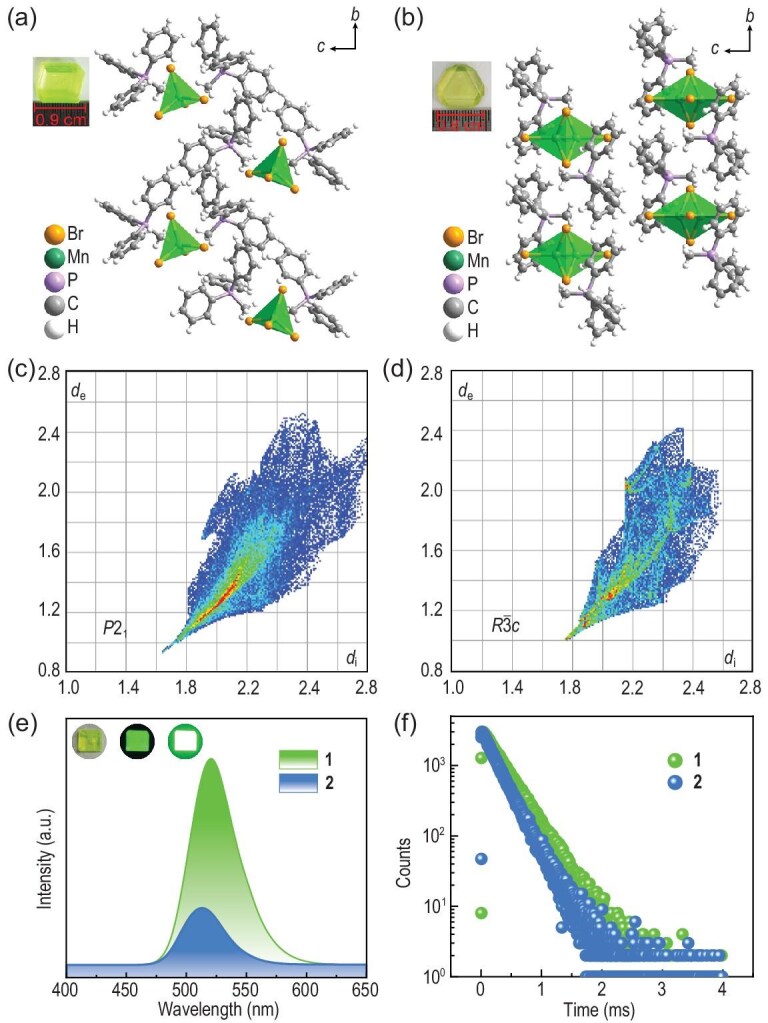
Structural characteristics and optical properties of **1** and **2**. (a, b) Crystal structures of **1** and **2**, with the upper left corner insets displaying photographs of single crystals for both. (c, d) The 2D fingerprints of **1** and **2**. (e) Luminescent spectra of **1** and **2**. Inset: The optical images of the crystals under sunlight (left) and a 365 nm UV lamp (middle, UV light on the crystal side; right, UV light on the crystal top). (f) Time-resolved emission spectra of **1** and **2**.

Illustrated in the upper left inset of Fig. 1a and b, both crystals can achieve sizes up to centimeters under ambient conditions, providing a crucial basis for ML tests and potential corresponding applications. The determination of crystal structures was accomplished through single crystal X-ray diffraction (XRD), revealing that they crystallize in the monoclinic space group *P*2_1_ for polymorph **1** [29,30] and trigonal space group *R*${\mathrm{\bar{3}}}$*c* for polymorph **2**, respectively (Fig. 1a and b). For the structure of polymorph **2**, it was previously reported to be *R*3*c* [31], which is slightly lower than the centrosymmetric space group solved from our data. Additional and detailed crystallographic data are provided in Table S1. Powder X-ray diffraction (PXRD) (Fig. S1) was conducted, and the refined results were compared with the simulated patterns, demonstrating their excellent phase purity. The independent segments of the unit cell for **1** and **2** comprise one [MnBr_4_]^2−^ anion and two C_19_H_18_P^+^ cations, loosely connected through electrostatic interactions, forming a characteristic 0D structure. However, the two Mn^2+^ ions in **2** exhibit positional disorder, with a $\frac{1}{2}$ occupancy within the [MnBr_4_]^2−^ tetrahedron. The Mn–Br bond lengths in **1** range from 2.468 to 2.527 Å and the Br–Mn–Br bond angles vary from 107.31 to 112.44°, resulting in a slightly twisted tetrahedron [31].

Hirshfeld surfaces and corresponding 2D fingerprints were obtained to clarify the intermolecular interactions between (C_19_H_18_P)^+^ cations and [MnBr_4_]^2−^ tetrahedra within the asymmetric unit [32]. In the 2D fingerprints (Fig. 1c and d), blue, green, and red points signify progressively strengthened interactions. Moreover, *d*_i_ and *d*_e_ represent the distances between the chosen [MnBr_4_]^2−^ tetrahedron and the surrounding atoms inside and outside the Hirshfeld surface. The calculated (*d*_i_, *d*_e_)_min_ values for **1** and **2** are (1.6400, 0.9334) and (1.7521, 1.0145), respectively, indicating shorter distances between the organic cation and [MnBr_4_]^2−^ tetrahedron in **1**. Simultaneously, the 2D fingerprints reveal abundant and wide red, green, and blue points in **1**, in contrast to their dispersed and subtle presence in **2**. This distinction is further evident in the Hirshfeld surfaces depicted in Fig. S2. Blue, white, and red regions correspond to distances longer, equal to, and shorter than the van der Waals distances, respectively. Notably, four red areas are observed in **1**, while three are present in **2**, with the distribution of blue areas being more extensive in **2**. These findings collectively indicate that the interactions between organic and inorganic components in **1** are stronger, resulting in a more stable structure.

To characterize the optical properties of **1** and **2**, their luminescence spectra were recorded as depicted in Fig. 1e. The single crystal of **1** shows bright green emission under UV light irradiation, as shown in the inset, which is typical for tetrahedrally coordinated Mn^2+^ ions and attributed to the ^4^T_1_(^4^G)–^6^A_1_(^6^S) transition [33,34]. The emission spectrum of **1** shows a peak centred at 520.6 nm and a full width at half maxima (FWHM) of 47 nm. In contrast, **2** exhibits a narrower emission centred at 513 nm with an FWHM of 42 nm, indicating that both compounds can achieve green emission with higher color purity. The average lifetime, based on double-exponential fitting (Fig. 1f), is 320 μs for **1** and 259 μs for **2**, indicative of the phosphorescent nature of the emission. The stronger interactions between the organic cation and [MnBr_4_]^2−^ in **1** give rise to several property differences, including, but not limited to the luminescence quantum yield, measured at 92.5% for **1** and 55.0% for **2**. The discernible contrast in luminescence quantum yield values is attributed to the enhanced interactions impeding the movement of [MnBr_4_]^2−^ and reducing energy loss, thereby improving luminescence quantum yield [28,35].

**Figure 2.**
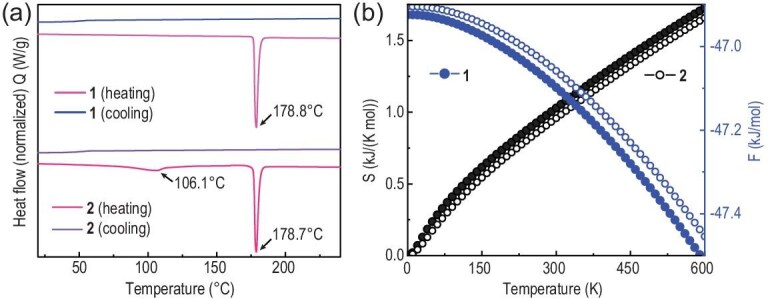
Thermodynamic tests and calculations. (a) DSC curves of **1** and **2** at a rate of 10°C/min. (b) The entropy of the phonons and the free energy of **1** and **2**.

DSC was employed to investigate the crystallization behavior of the two crystals (Fig. 2a). During the heating procedure at a rate of 10°C/min, the melting points of **1** and **2** were determined to be 178.8 and 178.7°C, respectively. No crystallization peak was observed during the cooling process, since a glassy state is formed after melting [36]. Surprisingly, at 106.1°C, a phase transition point of *R*${\mathrm{\bar{3}}}$*c* was detected, and subsequent XRD test results indicated that the *R*${\mathrm{\bar{3}}}$*c* transformed into the *P*2_1_ phase. Subsequently, the crystal structure of *R*${\mathrm{\bar{3}}}$*c* gradually changed into the structure of *P*2_1_ when the temperature reached 90°C, as confirmed by variable temperature XRD results (Fig. S3). Crystal **2** with *R*${\mathrm{\bar{3}}}$*c* is structurally unstable at ambient conditions, and it spontaneously transforms into Crystal **1** with *P*2_1_ after several days in air as confirmed by time-depedent PXRD and crystal morphology (Fig. S4). To delve deeper into the thermodynamic stability of **1** and **2**, an exploration of temperature-dependent thermodynamic properties and phase stabilities were conducted. The temperature-dependent total free energies (*F*) of the systems and entropy (*S*) of the phonons are shown in Fig. 2b. Consequently, **1** emerges as the thermodynamically stable phase, characterized by lower free energy and higher entropy. This finding aligns with the results obtained from the Hirshfeld surfaces and corresponding 2D fingerprint.

**Figure 3.**
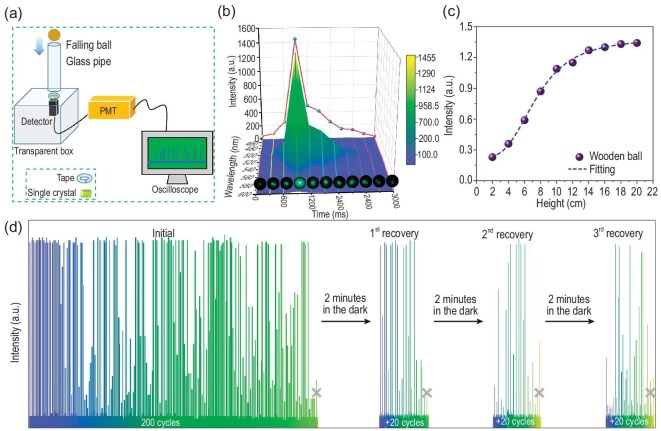
Mechanoluminescence features of **1.** (a) Schematic representation of the self-built drop tower apparatus. (b) Dynamic spectra of the FML process of crystal **1**, inset: the corresponding optical images of the crystal. (c) A curve illustrating the relationship between the height of the ball drop and ML intensity. The dashed line is a guide to the eye. (d) Fatigue resistance test and 3 cycles of self-recovery experiment with two-minute intervals.

The ML emission spectrum was measured using a self-built drop tower apparatus as shown in Fig. 3a and Fig. S5 [37,38]. In this setup, a wooden ball with an 18 mm diameter and a weight of 2 g was positioned at the top centre of the glass pipe. As the ball descended, it applied a mechanical force to the crystal placed above a transparent plexiglass box. The resulting optical signals were captured by a detector positioned underneath. The signals were then amplified and recorded using a photomultiplier tube (PMT) and an oscilloscope, respectively.

Without any treatment, crystal **1** undergoes fracturing upon impact, leading to FML, which is reminiscent of the phenomenon reported in an early pioneering work [22]. Fig. 3b illustrates the FML emission spectra and corresponding snapshots measured at intervals of 300 ms throughout the entire 3-second FML process. Throughout this duration, the FML intensity steadily increases, reaching its peak at 900 ms, and then decreases gradually. The pronounced green emissions, centred around 519 nm, closely align with the luminescence spectra, indicating that the FML results from Mn^2+^ transitions from the ^4^T_1_(^4^G) level to the lower ^6^A_1_(^6^S) level [11].

To investigate the EML process, the crystals of **1** were wrapped in a 2-mm-thick tape as a precaution against breakage. To ascertain how the EML intensity fluctuates with different impacts, the ball drop height was varied from 2 to 20 cm, corresponding to impact energies ranging from 0.04 to 0.4 J. A transparent crystal of high quality was chosen for this investigation, as it emitted the most EML, facilitating the measurement of EML for lower impact energies. The results of this test are shown in Fig. 3c. The minimum detectable height for EML was determined to be 2 cm, showcasing a promising potential for sensitive applications. It is evident that the energy at impact significantly influences the amount of EML emitted. The intensity of EML exhibits nearly linear growth with the height of the ball when it is lower than 10 cm, after which the growth gradually decelerates. The fatigue resistance of **1** was depicted in Fig. 3d (left side), providing an assessment of its potential for device-based applications. When the balls were dropped from the same height, the EML occasionally weakened but did not completely vanish until after 200 cycles. An electric toothbrush with a high vibration frequency, commonly used in daily life, was employed as a stimulation source due to the time constraints imposed by the repeated falling of the ball. The crystal, still wrapped in tape, was positioned beneath the head of an electric toothbrush. The toothbrush vibrated at a frequency of 550 Hz for 2 minutes. Subsequently, the luminescence performance was tested using the same drop tower equipment. The crystal underwent three consecutive falling-ball tests to ensure experiment accuracy, and Fig. S6 illustrates the results. It was observed that there was minimal change in voltage during the three successive falling-ball tests, and the crystal retained its EML activity even after 30 minutes of stimulation with the electric toothbrush. The presented data indicates that the crystal exhibits notable fatigue resistance, suggesting promising possibilities for practical application.

**Figure 4.**
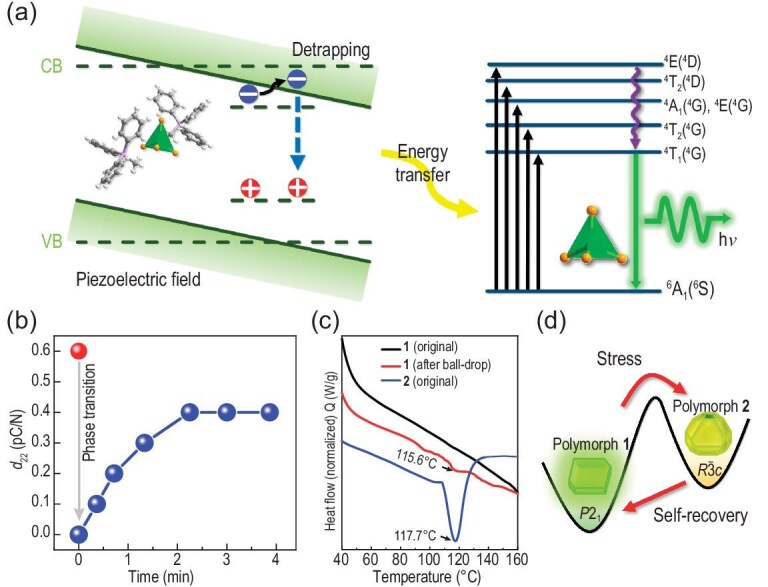
Proposed working mechanism of FML and self-recoverable EML. (a) Schematic representation of the ML mechanism. (b) Variation in the piezoelectric coefficient (*d*_22_) during fatigue and recovery. (c) DSC curves at a rate of 35°C/min. (d) The mechanism of fatigue and self-recovery.

The self-recovery ability was examined by subjecting crystal **1** to impact after it entered the fatigue period, and then allowing it to rest in the dark for 2 minutes. Astonishingly, EML was observed once again. After nearly 20 consecutive stimulations, the crystal consistently showed EML intensity before entering the second fatigue period. Subsequently, when shielded from light for two minutes, the ball fell, and EML recovered repeatedly. The entire process was repeated six times (Fig. 3d, right side; Fig. S7). The findings indicate that the crystal possesses an excellent self-recovery ability, once it reaches the luminescent fatigue stage, a brief period of rest in the dark is sufficient for it to resume emitting EML. The FML and EML processes are explained through the piezoelectric effect and the energy band tilt theory [13,39]. In a crystal with a non-centrosymmetric structure upon stress, the piezoelectric polarization causes the separation of charge carriers (Fig. 4a). These separated electrons and holes get snared by traps below the conduction band (CB) and above the valence band (VB), respectively. The band structure undergoes tilting under the internal piezoelectric potential. Because of the tilt, the trap depth becomes shallower, leading to the release and recombination of trapped charge carriers. Subsequently, the energy is transferred to the luminescent centre Mn^2+^. As Mn^2+^ cation returns to the ground state ^6^A_1_(^6^S) from the excited state ^4^T_1_(^4^G), a non-radiative transition occurs, resulting in green luminescence. The attenuation of piezoelectric luminescence can be attributed to the depletion of desorption electrons.

To understand the underlying mechanism of the self-recovery EML, piezoelectric and DSC measurements were conducted on the crystals of polymorph **1** before and after the fatigue resistance tests. In Fig. 4b, the piezoelectric coefficient *d*_22_ was found to be 0.6 pC/N for a crystal before undergoing mechanical treatment. The *d*_22_ was computed through DFT calculations, and the results showed that the *d*_22_ of **1** is 0.63 pC/N, in good agreement with the measured value, confirming the reliability of the experiment. However, *d*_22_ decreased to 0 when the crystal was mechanically stimulated for ∼200 times (Video S1), demonstrating the stress-induced phase transformation from *R*${\mathrm{\bar{3}}}$*c* to *P*2_1_. During recovery, *d*_22_ gradually increased from 0 to 0.1, 0.2, 0.3, and 0.4 pC/N with a 30-second time interval, then saturated after 2.5 minutes, nearly the same as the time scale for the crystal to recover (Fig. S8).

Additional DSC measurements were conducted at a rate of 35°C/min to address any potential limitations in capturing the material behavior during the fatigue period. The obtained results, illustrated in Fig. 4c, reveal an endothermic peak at 115.6°C in comparison to the unstressed crystal, closely aligning with the phase transition point of the trigonal nonpiezoelectric polymorph **2** under identical test conditions. To validate the accuracy of this deduction, an extensive series of tests were undertaken, and the results are shown in Fig. S9. These results strongly suggest that the fatigue of the EML is attributed to the monoclinic to trigonal phase transition, as highlighted in Fig. 4d. Given the energetically unfavorable nature of the trigonal phase **2**, it promptly reverts back to the piezoelectric polymorph **1**, elucidating the observed self-recovery phenomenon.

It is noteworthy that polymorph **1** exhibits a responsive behavior to common mechanical forces encountered in daily life. Figure S10a shows that the FML manifests stimulation when crystals of polymorph **1** are positioned in a glass bottle subjected to a flow of N_2_ introduced through a gas gun stressed to 0.5 MPa. Furthermore, even the polycrystalline films of **1** demonstrated exceptionally sensitive FML. Scratching or puncturing the polycrystalline films, formed through melting and recrystallization, results in the emission of a vibrant green light (Fig. S10b). The crystals of **1** display responsiveness to a range of mechanical stimuli, including squeezing, pressing, finger bending and wrist bending (Fig. S10c–f). This responsiveness indicates crystals of polymorph **1** are promising materials for sensing human movement. To facilitate operational procedures and protect the crystal from fractures, the single crystal is encapsulated with UV glue as shown in Fig. S11. Considering the self-recovery virtue, polymorph **1** could also have the potential to eliminate the problems encountered by traditional ML materials used in biomedicine. These materials act as ultrasound-responsive light sources to image local tissues but need to be repeatedly charged which is challenging. The self-recoverable polymorph **1** could effectively overcome this shortcoming to serve as next-generation materials for optogenetics [40–42]. Therefore, polymorph **1** characterized by excellent EML properties, emerges as a promising candidate for practical applications.

## CONCLUSIONS

In summary, two distinct types of OIMH crystals with varying phases were prepared through a solution-based method. Crystal **1**, crystallized in the *P*2_1_ space group, exhibits enhanced structural stability compared to crystal **2**, along with exceptional optical properties, high luminescence quantum yield and narrow FWHM. The distinctive non-centrosymmetric piezoelectric space group of crystal **1**, accompanied by piezoelectric fields and tilted energy bands under stress, gives rise to FML characteristics and strong fatigue resistance of EML, allowing for repetitive stress cycles numbering in the hundreds. The swift transition between the piezoelectric and non-piezoelectric space groups in crystals **1** and **2** enables the repetitive self-recoverable EML. The excellent ML and cycling properties of these 0D OIMH metal manganese halides position them with broad application prospects. This discovery and study of the phase transition during self-recovery contribute novel insights to the mechanical explanation of ML.

## Supplementary Material

nwae372_Supplemental_Files

## References

[1] Jha P, Chandra BP. Survey of the literature on mechanoluminescence from 1605 to 2013. Luminescence 2014; 29: 977–93. 10.1002/bio.2647 24753157

[2] Xie Y, Li Z. Triboluminescence: recalling interest and new aspects. Chem 2018; 4: 943–71. 10.1016/j.chempr.2018.01.001

[3] Zhang J-C, Wang X, Marriott G et al. Trap-controlled mechanoluminescent materials. Prog Mater Sci 2019; 103: 678–742. 10.1016/j.pmatsci.2019.02.001

[4] Chandra VK, Chandra BP, Jha P. Strong luminescence induced by elastic deformation of piezoelectric crystals. Appl Phys Lett 2013; 102: 241105. 10.1063/1.4811160

[5] Xu CN, Watanabe T, Akiyama M et al. Direct view of stress distribution in solid by mechanoluminescence. Appl Phys Lett 1999; 74: 2414–6. 10.1063/1.123865

[6] Xu CN, Watanabe T, Akiyama M et al. Artificial skin to sense mechanical stress by visible light emission. Appl Phys Lett 1999; 74: 1236–8. 10.1063/1.123510

[7] Zhao J, Chi Z, Zhang Y et al. Recent progress in the mechanofluorochromism of cyanoethylene derivatives with aggregation-induced emission. J Mater Chem C 2018; 6: 6327–53. 10.1039/C8TC01648H

[8] Liu S, Zheng Y, Peng D et al. Near-Infrared mechanoluminescence of Cr^3+^ doped gallate spinel and magnetoplumbite smart materials. Adv Funct Materials 2023; 33: 2209275. 10.1002/adfm.202209275

[9] Zhao Z, Zhang H, Lam JWY et al. Aggregation-induced emission: new vistas at the aggregate level. Angew Chem Int Ed 2020; 59: 9888–907. 10.1002/anie.201916729 32048428

[10] Chandra VK, Chandra BP, Jha P. Self-recovery of mechanoluminescence in ZnS:cu and ZnS:mn phosphors by trapping of drifting charge carriers. Appl Phys Lett 2013; 103: 161113. 10.1063/1.4825360

[11] Zhou T, Chen H, Guo J et al. Unrevealing temporal mechanoluminescence behaviors at high frequency via piezoelectric actuation. Small 2023; 19: 2207089. 10.1002/smll.202207089 36507549

[12] Zhuang Y, Xie R-J. Mechanoluminescence rebrightening the prospects of stress sensing: a review. Adv Mater 2021; 33: 2005925. 10.1002/adma.202005925 33786872

[13] Wang X, Zhang H, Yu R et al. Dynamic pressure mapping of personalized handwriting by a flexible sensor matrix based on the mechanoluminescence process. Adv Mater 2015; 27: 2324–31. 10.1002/adma.201405826 25711141

[14] Luo J, Ren B, Zhang X et al. Modulating smart mechanoluminescent phosphors for multistimuli responsive optical wood. Adv Sci 2024; 11: 2305066. 10.1002/advs.202305066 PMC10767394 37939290

[15] Huang B . Energy harvesting and conversion mechanisms for intrinsic upconverted mechano-persistent luminescence in CaZnOS. Phys Chem Chem Phys 2016; 18: 25946–74. 10.1039/C6CP04706H 27711528

[16] Feng A, Smet PF. A review of mechanoluminescence in inorganic solids: compounds, mechanisms, models and applications. Materials 2018; 11: 484. 10.3390/ma11040484 29570650 PMC5951330

[17] Wu C, Zeng S, Wang Z et al. Efficient mechanoluminescent elastomers for dual-responsive anticounterfeiting device and stretching/strain sensor with multimode sensibility. Adv Funct Mater 2018; 28: 1803168. 10.1002/adfm.201803168

[18] Yang J, Gao X, Xie Z et al. Elucidating the excited state of mechanoluminescence in organic luminogens with room-temperature phosphorescence. Angew Chem Int Ed 2017; 56: 15299–303. 10.1002/anie.201708119 28981197

[19] Zhang Q, Wang Y, Braunstein P et al. Construction of olefinic coordination polymer single crystal platforms: precise organic synthesis, in situ exploration of reaction mechanisms and beyond. Chem Soc Rev 2024; 53: 5227–63. 10.1039/D3CS01050C 38597808

[20] Chen Y, Yang Z, Wu X-Y et al. Dielectric anisotropy of the single crystal of isopropylviologen copper(I) triiodide. Phys Chem Chem Phys 2011; 13: 10781–6. 10.1039/c1cp20422j 21547321

[21] Chen Y, Wang Z-O, Yang Z et al. Unique assembly of low-dimensional viologen iodoplumbates and their improved semiconducting properties. Dalton Trans 2010; 39: 9476–9. 10.1039/c0dt00757a 20838680

[22] Balsamy S, Natarajan P, Vedalakshmi R et al. Triboluminescence and vapor-induced phase transitions in the solids of methyltriphenylphosphonium tetrahalomanganate(II) complexes. Inorg Chem 2014; 53: 6054–9. 10.1021/ic500400y 24899549

[23] He X, Zheng Y, Luo Z et al. Bright circularly polarized mechanoluminescence from 0D hybrid manganese halides. Adv Mater 2024; 36: 2309906. 10.1002/adma.202309906 38228314

[24] Liu X, Ge C, Yang Z et al. Guanidine-templated manganese halides single crystals toward efficient mechanoluminescence and photoluminescence by supramolecular interactions modulation. Adv Opt Mater 2021; 9: 2100862. 10.1002/adom.202100862

[25] Zhou G, Liu Z, Molokeev MS et al. Manipulation of Cl/Br transmutation in zero-dimensional Mn^2+^-based metal halides toward tunable photoluminescence and thermal quenching behaviors. J Mater Chem C 2021; 9: 2047–53. 10.1039/D0TC05137C

[26] Guo TM, Gong YJ, Li ZG et al. A new hybrid lead-free metal halide piezoelectric for energy harvesting and human motion sensing. Small 2022; 18: 2103829. 10.1002/smll.202103829 34825468

[27] Chen J, Zhang Q, Zheng F-K et al. Intense photo- and tribo-luminescence of three tetrahedral manganese(II) dihalides with chelating bidentate phosphine oxide ligand. Dalton Trans 2015; 44: 3289–94. 10.1039/C4DT03694H 25597698

[28] Mao L, Guo P, Wang S et al. Design principles for enhancing photoluminescence quantum yield in hybrid manganese bromides. J Am Chem Soc 2020; 142: 13582–9. 10.1021/jacs.0c06039 32693585

[29] Qin Y, She P, Guo S et al. Structural manipulation and triboluminescence of tetrahalomanganese(II) complexes. Acta Phys -Chim Sin 2020; 36: 1907078. 10.3866/PKU.WHXB201907078

[30] Lin J, Zhang M, Sun N et al. Narrowing the band of green emission in manganese hybrids by reducing the hydrogen bond strength and structural distortion. J Mater Chem C 2022; 10: 16773–80. 10.1039/D2TC03481F

[31] Zhang W, Sui P, Zheng W et al. Pseudo-2D layered organic-inorganic manganese bromide with a near-unity photoluminescence quantum yield for white light-emitting diode and X-ray scintillator. Angew Chem Int Ed 2023; 62: e202309230. 10.1002/anie.202309230 37747789

[32] Spackman MA, Jayatilaka D. Hirshfeld surface analysis. CrystEngComm 2009; 11: 19–32. 10.1039/B818330A

[33] Jana A, Zhumagali S, Ba Q et al. Direct emission from quartet excited states triggered by upconversion phenomena in solid-phase synthesized fluorescent lead-free organic–inorganic hybrid compounds. J Mater Chem A 2019; 7: 26504–12. 10.1039/C9TA08268A

[34] Morad V, Cherniukh I, Pottschacher L et al. Manganese(II) in tetrahedral halide environment: factors governing bright green luminescence. Chem Mater 2019; 31: 10161–9. 10.1021/acs.chemmater.9b03782 32952294 PMC7493303

[35] Mao L, Chen J, Vishnoi P et al. The renaissance of functional hybrid transition-metal halides. Acc Mater Res 2022; 3: 439–48. 10.1021/accountsmr.1c00270

[36] Li B, Xu Y. Zero-dimensional luminescent metal halide hybrids enabling bulk transparent medium as large-area X-ray scintillators. Adv Opt Mater 2022; 10: 2102793. 10.1002/adom.202102793

[37] Fontenot RS, Allison SW, Lynch KJ et al. Mechanical, spectral, and luminescence properties of ZnS:mn doped PDMS. J Lumin 2016; 170: 194–9. 10.1016/j.jlumin.2015.10.047

[38] Fontenot RS, Hollerman WA, Bhat KN et al. Effects of added uranium on the triboluminescent properties of europium dibenzoylmethide triethylammonium. J Lumin 2013; 134: 477–82. 10.1016/j.jlumin.2012.07.042

[39] Peng D, Jiang Y, Huang B et al. A ZnS/CaZnOS heterojunction for efficient mechanical-to-optical energy conversion by conduction band offset. Adv Mater 2020; 32: 1907747. 10.1002/adma.201907747 32128925

[40] Wu X, Zhu X, Chong P et al. Sono-optogenetics facilitated by a circulation-delivered rechargeable light source for minimally invasive optogenetics. Proc Natl Acad Sci USA 2019; 116: 26332–42. 10.1073/pnas.1914387116 31811026 PMC6936518

[41] Yang F, Wu X, Cui H et al. Palette of rechargeable mechanoluminescent fluids produced by a biomineral-inspired suppressed dissolution approach. J Am Chem Soc 2022; 144: 18406–18. 10.1021/jacs.2c06724 36190898 PMC10519178

[42] Jiang S, Wu X, Yang F et al. Activation of mechanoluminescent nanotransducers by focused ultrasound enables light delivery to deep-seated tissue in vivo. Nat Protoc 2023; 18: 3787–820. 10.1038/s41596-023-00895-8 37914782 PMC11405139

