# Supplementary material for: Self-recoverable elastico mechanoluminescence of a hybrid metal halide crystal
Source: Natl Sci Rev. 2024 Oct 21;12(5):nwae372. doi: 10.1093/nsr/nwae372 (PMC11970250; doi:10.1093/nsr/nwae372)
Supplement: nwae372_Supplemental_Files [file nwae372_supplemental_files.zip › Supporting_Information.pdf]

Supporting Information

Self-Recoverable Elastico Mechanoluminescence of a Hybrid Metal Halide Crystal

Tian-Yi Yang[1,3], Si-Nuo Li[1,3], Hai-Sheng Chen[2], Zi-Ying Li[1], Zhi-Gang Li[1], Rui Feng[1], Fei-Fei Gao[1], Ying Zhang[1], Yi-Ming Liu[1], Yang Zhang*[,2], Wei-Wei Liu[2], Wei Li*[,1], Xian-He Bu[1]

1. *School of Materials Science and Engineering & Tianjin Key Laboratory of Metal and Molecule-Based Material Chemistry, Nankai University, Tianjin 300350, China.*
2. *Institute of Modern Optics & Tianjin Key Laboratory of Micro-Scale Optical Information Science and Technology, Nankai University, Tianjin, 300350, China*
3. *These authors contributed equally: Tian-Yi Yang, Si-Nuo Li.*

Materials. Manganese bromide ($MnBr_2$, 98.0%) and methyltriphenylphosphonium bromide ($C_{19}H_{18}BrP$, 98.0%), CH_3CH_2OH and hydrobromic acid (HBr, 48 wt.% in H_2O) were used as purchased for synthesis.

Synthesis. $C_{19}H_{18}BrP$ (0.7144 g, 2 mmol) and $MnBr_2$ (0.2148 g, 1 mmol) were added into a pyrex bottle containing CH_3CH_2OH (5 mL) and HBr (1.5 mL). The colorless and transparent solution was concentrated after three days of evaporation at room temperature. After washed by ethanol, bright green crystals of **1** were obtained after filtration. All of the crystals were dried in an oven at 50 ℃ for 6 hours before use. The synthesis procedure of **2** is almost the same as phase **1**, except the temperature was heated to 50 ℃ for three days. Then yellowish-green crystals **2** were obtained by similar treatment.

Single-crystal X-ray diffraction data collection and refinement. Single crystal X-ray diffraction experiment was performed on Rigaku XtaLAB PRO MM007 DW at 100 K, with a Hybrid Pixel Array CCD Detector (Cu *K*α λ = 1.5418 Å). A good quality crystal was selected to collect the single crystal data at 100 K. Subsequently, data collection and structural refinement were performed using the Rigaku CrysAlisPro 1.171.40.84a (Rigaku OD, 2020) and Olex2 software package.[1] The crystal data and structure refinement parameters are summarized in Table S1.

Powder X-ray diffraction. Powder X-ray diffraction patterns in a range of 2θ from 3 °to 50 ° were collected on a Rigaku MiniFlex600 diffractometer (40 kV, 15 mA) equipped with a Cu (*K*α, λ = 1.5418 Å) target tube. Le Bail whole profile fitting was carried out using the Unit Cell software and TOPAS program.

Hirshfeld surfaces and corresponding 2D fingerprints calculation.
Crystal Explorer 17.5 software was used to perform Hirshfeld Surface simulation for **1** and **2** crystals to analyze the Internal interaction. The $[MnBr_4]^{2-}$ Hirshfeld Surface in **1** and **2** was simulated using CIF file obtained at 100 K, and the interactions of $[MnBr_4]^{2-}$ with organic cation $(C_{19}H_{18}P)^+$ in **1** and **2** were obtained.

The entropy of the phonons and the free energy calculations. In the calculations, the energy of the structure was calculated by using the PBEsol functional in the VASP package.[2] The cut off energy was set to 500 eV, and k-point mesh sampling density with a

target length cut off of 25 Å, DFT-D3 Grimme correlation was adopted to account for dispersion effects. Before the phonon calculations, the structures were completely optimized (lattice constants and ionic positions) until the forces on all atoms were less than 10^{-3} eV/Å. The Mn^{2+} ions in **1** and **2** were regarded as a high-spin state, and the spin orientations were allowed to relax automatically. Besides, the magnetic contribution of Mn^{2+} is trivial to the calculations and has not been taken into account. For lattice dynamics calculations, we used a $2 \times 2 \times 1$ supercell structure for **1** and $1 \times 1 \times 1$ for **2**. For phonon calculations, the dynamical matrix and phonon frequencies were obtained by using the PHONOPY package under the frozen phonon approximation.[3] The vibrational properties were calculated by using a Q-point mesh of resolution $25 \times 25 \times 25$.

Piezoelectric coefficient calculations.

The piezoelectric coefficients [d] were obtained by the formula: [d] = [e] × [s]. The elastic compliance constants [s] are equal to the inverse matrix of elastic stiffness constants c_{ij}. The c_{ij} constants were obtained by the stress-strain method using DFT. Specifically, the calculations were performed using a plane-wave basis set as implemented in the Vienna Ab initio Simulation Package (VASP).[4-6] Given the strict requirements for calculating elastic constants, we fully relaxed the atomic positions and cell parameters during the structural optimization process. The elastic constants were calculated by applying six finite lattice distortions with a step size of 0.015 Å. The total energy was converged to within 10^{-7} eV, and the residual forces on each atom were reduced to less than 0.005 eV/Å. The DFT-D3 method was used to account for the dispersion corrections. The piezoelectric stress constants [e] were calculated by using the density functional perturbation theory (DFPT) method.

Photoluminescence (PL) spectra and time-resolved PL spectra. PL signal was recorded by the Horiba LabRAM HR Evolution system (HORIBA Jobin Yvon S.A.S., France) and the wavelength of the excitation was 325 nm. Time-resolved PL was performed on Edinburgh FLS 980 spectrofluorometer with Xe lamp as excitation.

Powder XRD patterns collected during the phase transformation: Crystals were collected out of the mother liquor from a Pyrex bottle and dealt with as described in the synthesis method. These crystals were then exposed to air for varying durations before being ground for powder XRD examinations.

ML experiments and FML process dynamic spectra. All the ML experiments were performed through the home-made drop tower equipment. A 2 gram, 18 mm-diameter wooden ball falls from the top of a glass pipe 20 cm above the sample, and the signal is collected via a PMT and displayed on an oscilloscope. To prevent crystal fracture during the EML test, the crystal is wrapped in a layer of around 2 mm thick, colorless transparent tape made of polypropylene ester. FML process dynamic spectra were captured by QEPro High-Performance Spectrometer which can capture 200 sets of spectra in one minute. A single crystal was placed in the middle of the bottom glass slide, and another glass slide was used to cover the crystal, the spectrometer was used to capture the spectra when pressing the upper glass slide, meanwhile, continuous pictures were taken to obtain crystal photos and FML spectra.

Differential scanning calorimetry (DSC) test. TA DSC25 was used for obtaining the DSC curves. The test was executed in helium atmosphere, the test temperature range is from −20°C to 250 ℃, and the heating/cooling rate is 10 ℃/min. But in order to record the change of **1** following stress stimulation, the heating/cooling rate is raised to 35 ℃/min.

Piezoelectric coefficient measurements. To measure piezoelectric coefficient d_{22}, the quasi-static method (ZJ-3AN) was used on the crystals.

Data and materials availability. The single-crystal structures in this work have been reported in the previous literature (The Cambridge Crystallographic Data Center (CCDC) database: $[C_{19}H_{18}P]_2MnBr_4$: 2162994, space group $P2_1$; $[C_{19}H_{18}P]_2MnBr_4$: 2235118, space group $R3c$. Nevertheless, the data were recollected by us, and our crystallographic .cif files are provided in the supplementary files. It should be noted that the trigonal polymorph of our data has an $R\overline{3}c$ space group which has higher symmetry than the reported $R3c$ of structure 2235118.

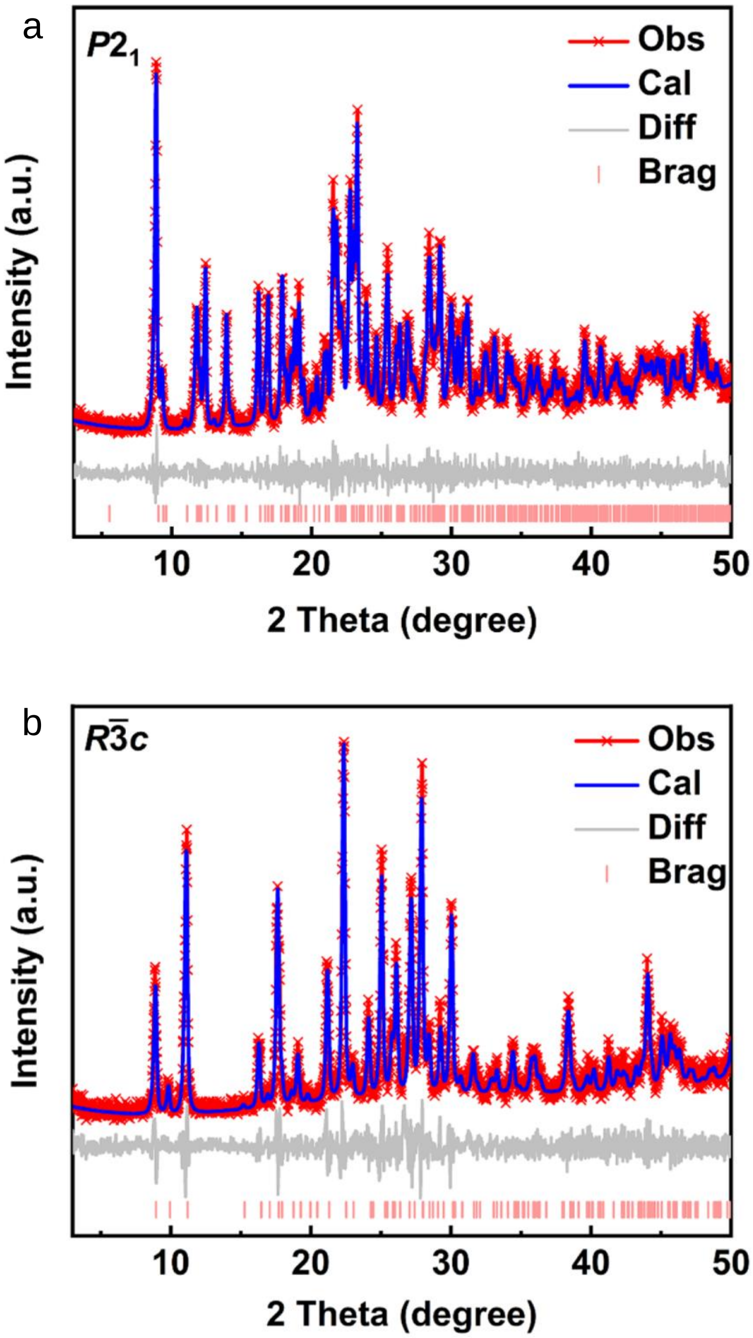

Figure S1. Two phases of $[C_{19}H_{18}P]_2MnBr_4$ powder XRD diffraction patterns obtained by Le-Bail fitting with Topas software. (a) $P2_1$; (b) $R\overline{3}c$. Red symbol-line: X-ray powder diffraction patterns at room temperature; Blue line: calculated profiles; Grey lines: the difference between experimental and calculated profiles. Rosy vertical mark: the position of the Bragg diffraction peaks.

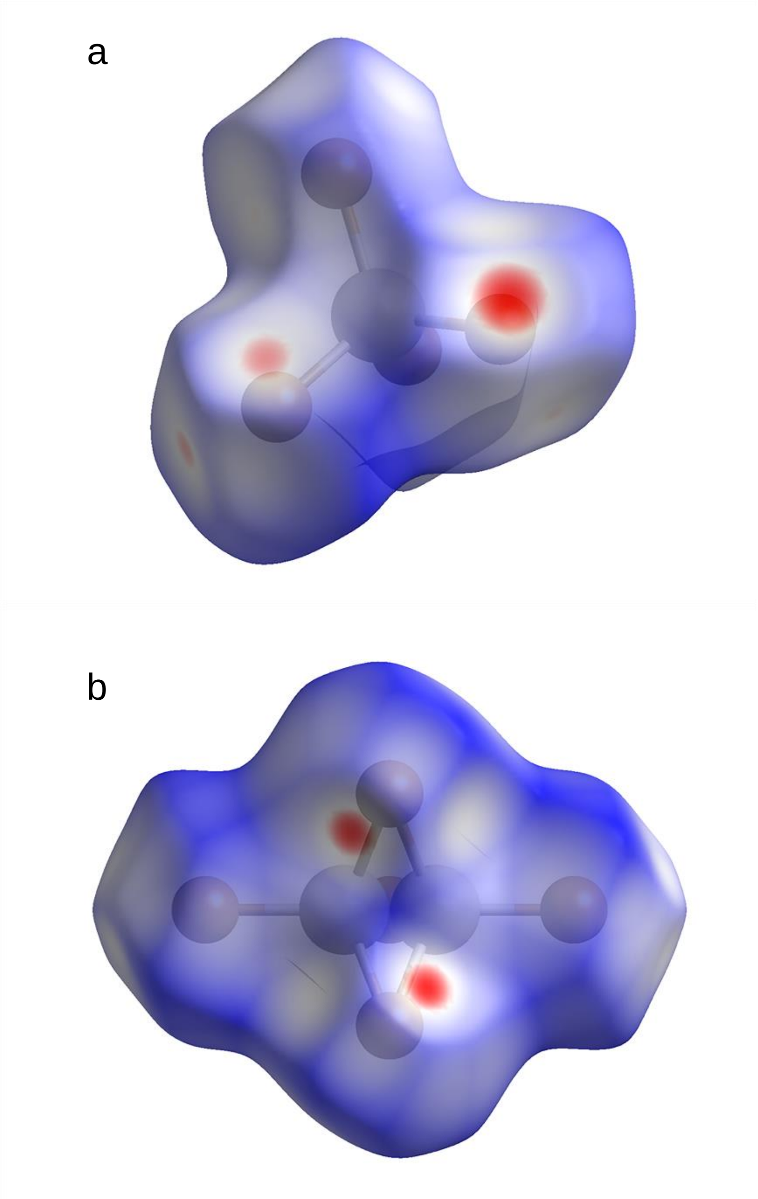

Figure S2. Hirshfeld surfaces of two independent $[MnBr_4]^{2-}$ in the asymmetric unit of $[C_{19}H_{18}P]_2MnBr_4$**.** (a) $P2_1$; (b) $R\bar{3}c$. Blue, white, and red represent the distances that are longer, equal to, and shorter than the van der Waals distances, respectively. Four red areas in **1** while three areas in **2** and the blue areas are mostly and widely distributed in **2**.

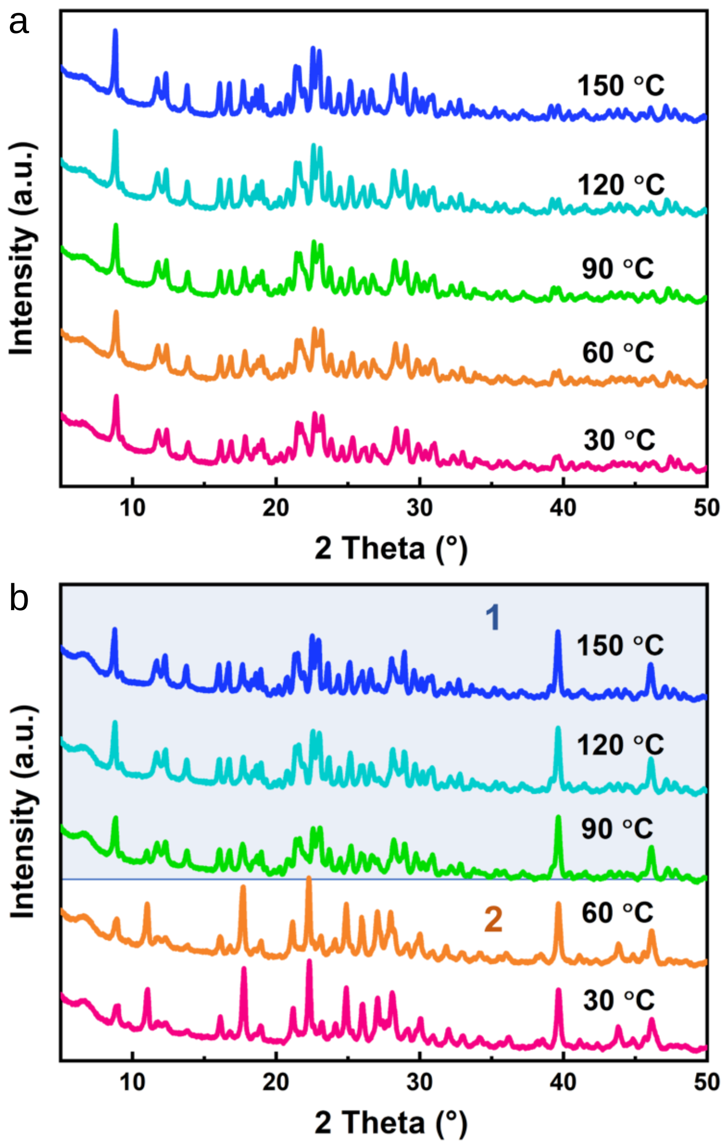

Figure S3. XRD patterns of (a) **1**, and (b) **2** at different temperatures.

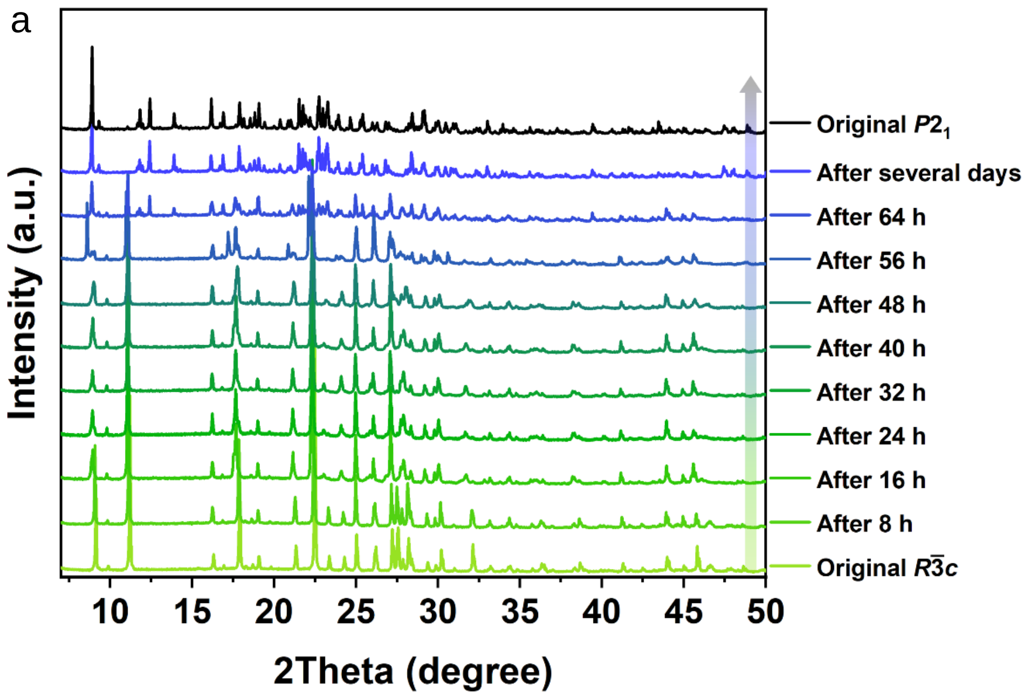

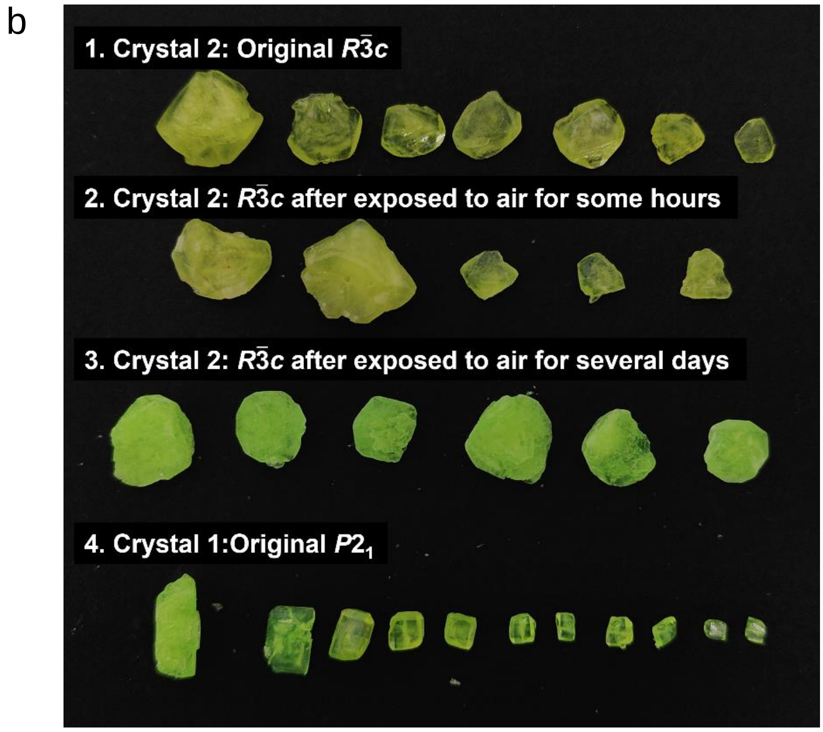

Figure S4. (a) The PXRD patterns collected during the phase transformation between the two polymorphs. (b) Photograph of Crystal **2** exposed to air and the gradual transformation into Crystal **1** over time.

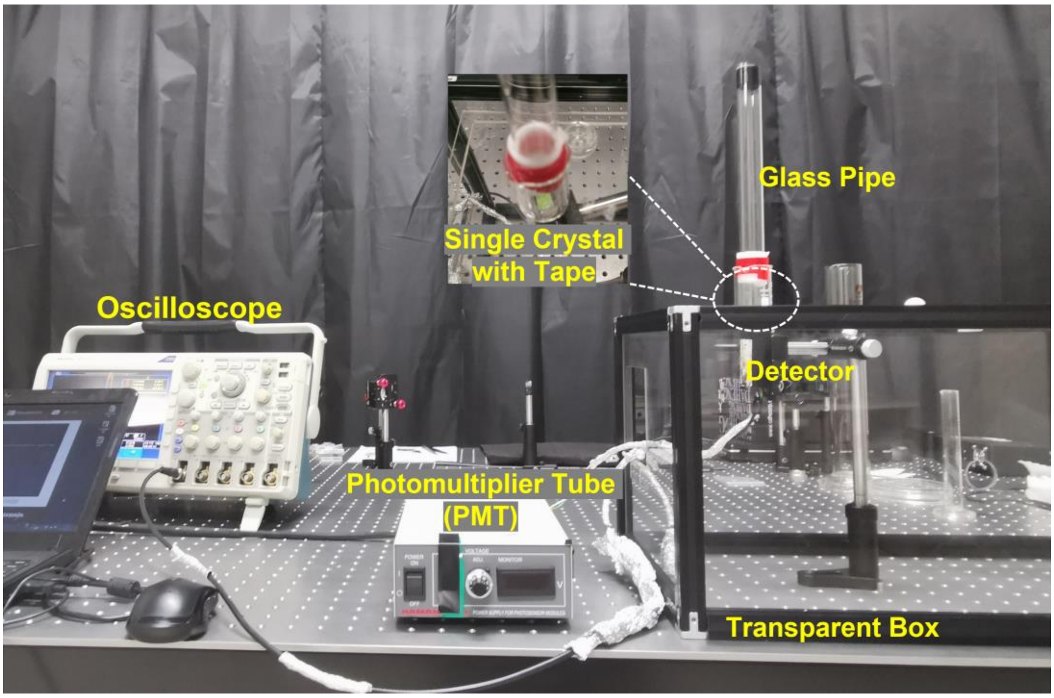

Figure S5. Photograph of the self-built drop tower apparatus.

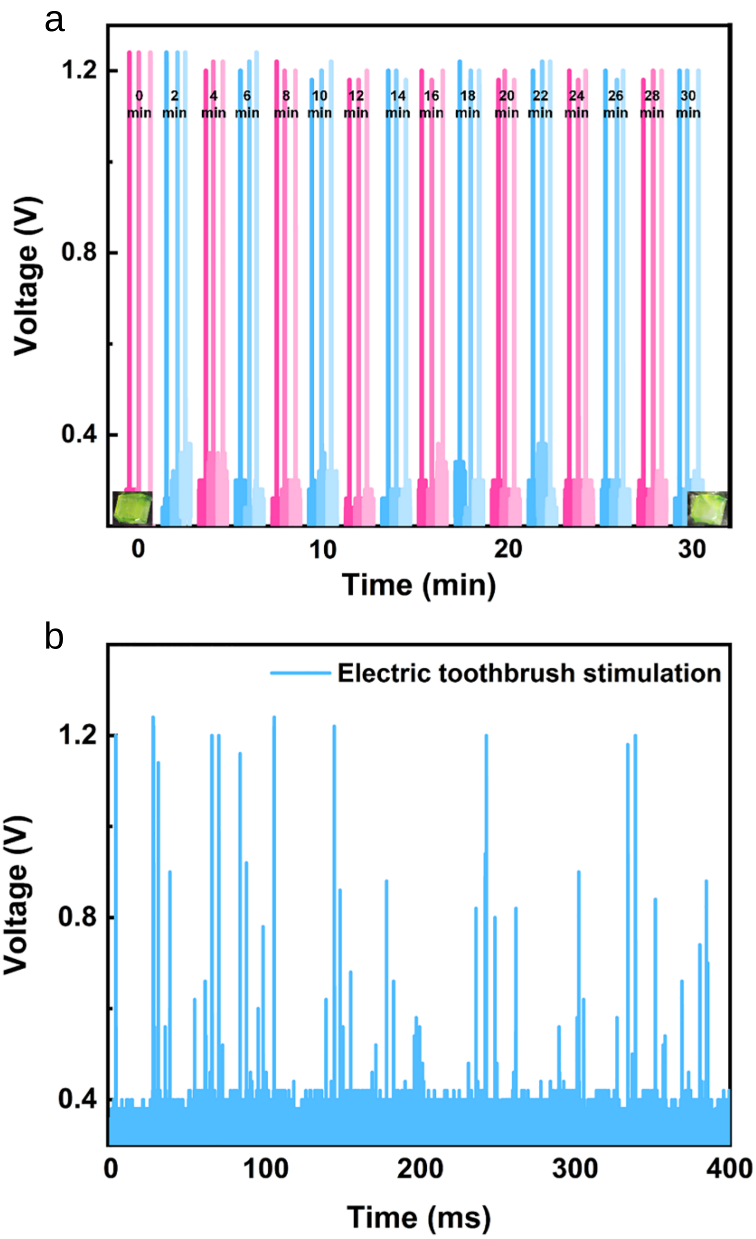

Figure S6. Fatigue resistance experiment. Electric toothbrush stimulus (standard model: 33000 times/min, frequency=550 Hz, 2 minutes stimulus: 66000 times) as the attenuation method and falling ball as the characterization method. (a) The falling ball is performed to verify the EML signal after every 2 minutes of toothbrush stimulus, (b) after 30 minutes of electric toothbrush stimulus (990000 times stimulus) EML signal still exists.

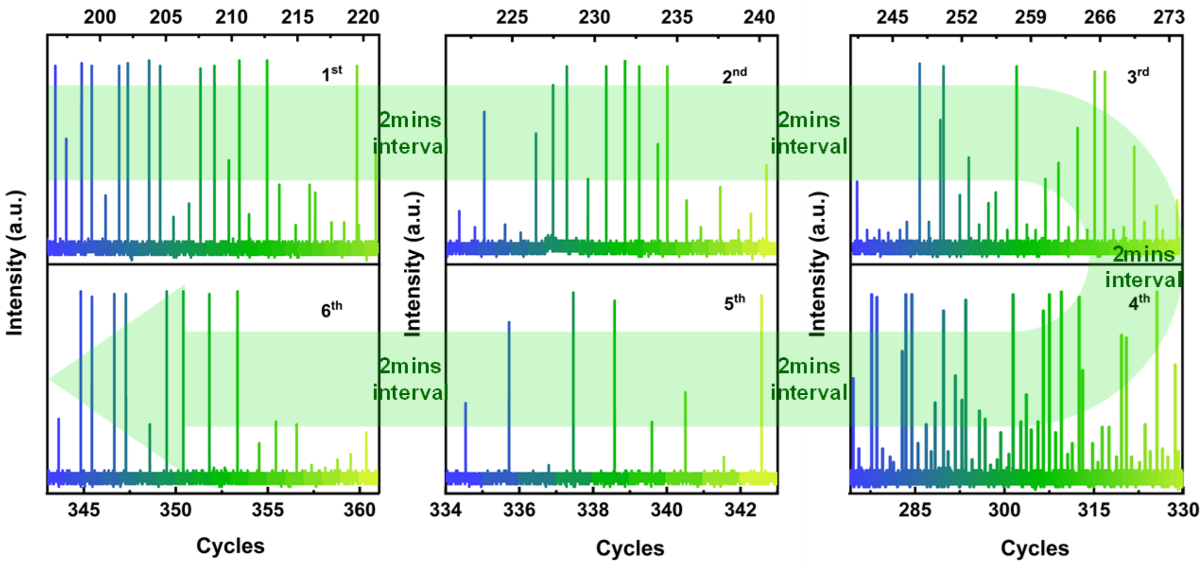

Figure S7. Six cycles of self-recovery experiment with two-minute intervals.

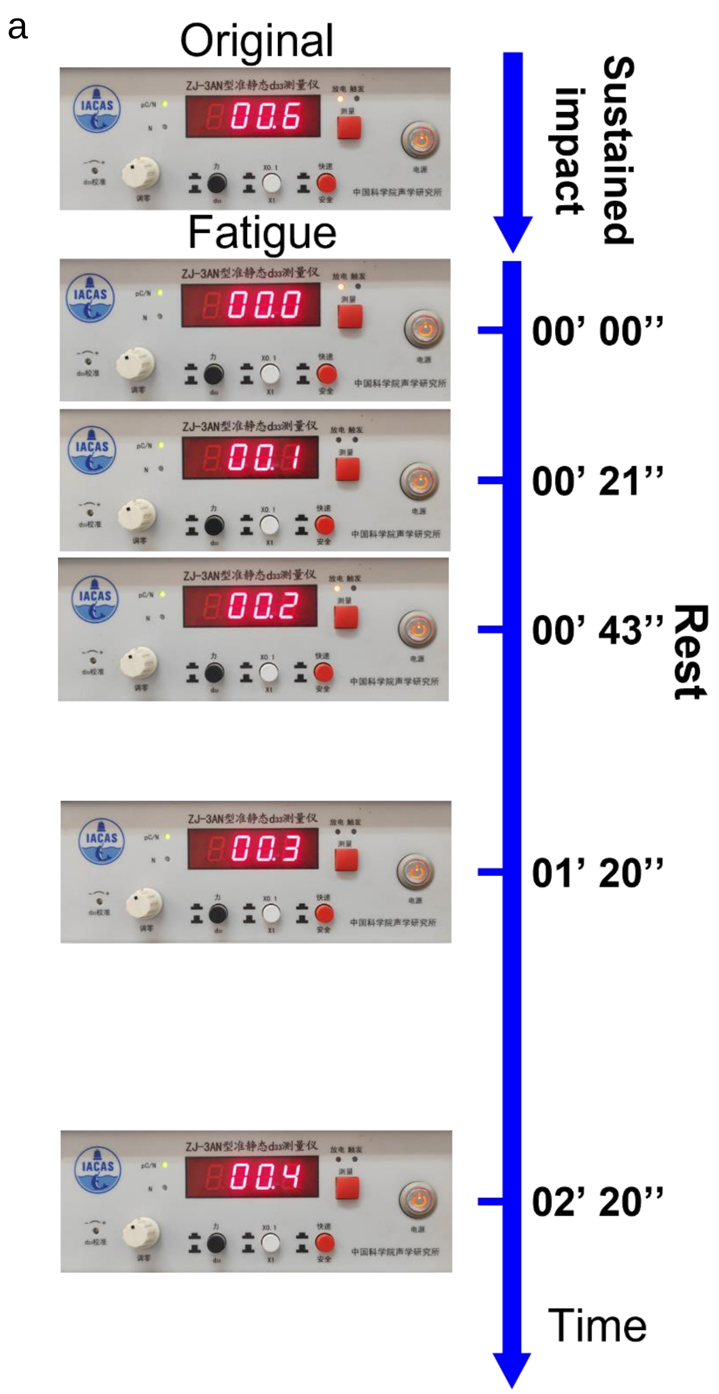
a
Original
Fatigue
Sustained impact
Rest
00’ 00’’
00’ 21’’
00’ 43’’
01’ 20’’
02’ 20’’
Time

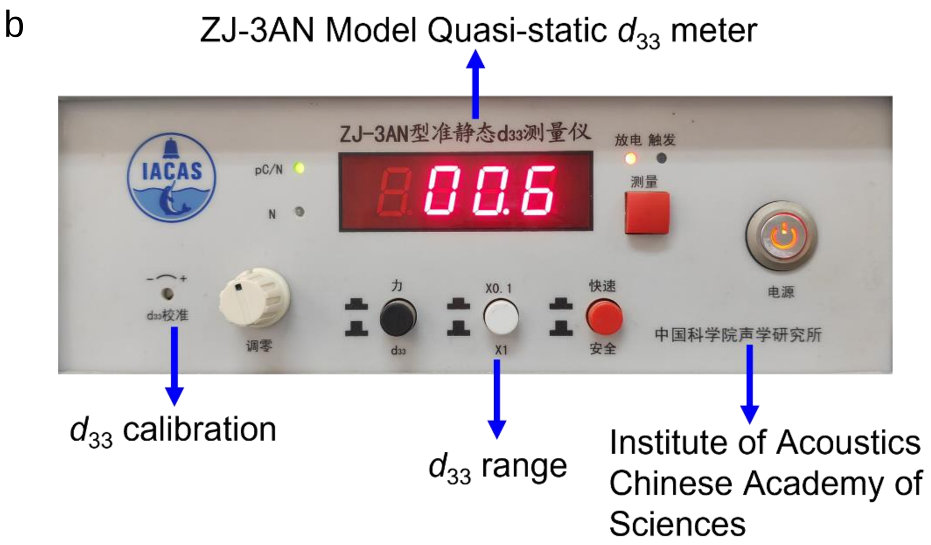

Figure S8. (a) Raw data of measured piezoelectric coefficient d_{22} for original, fatigue and gradual recovery. (b) Interface translation for the instruction of quasi-static piezoelectric meter. Piezoelectric coefficient d_{22} can be read directly from the quasi-static piezoelectric meter.

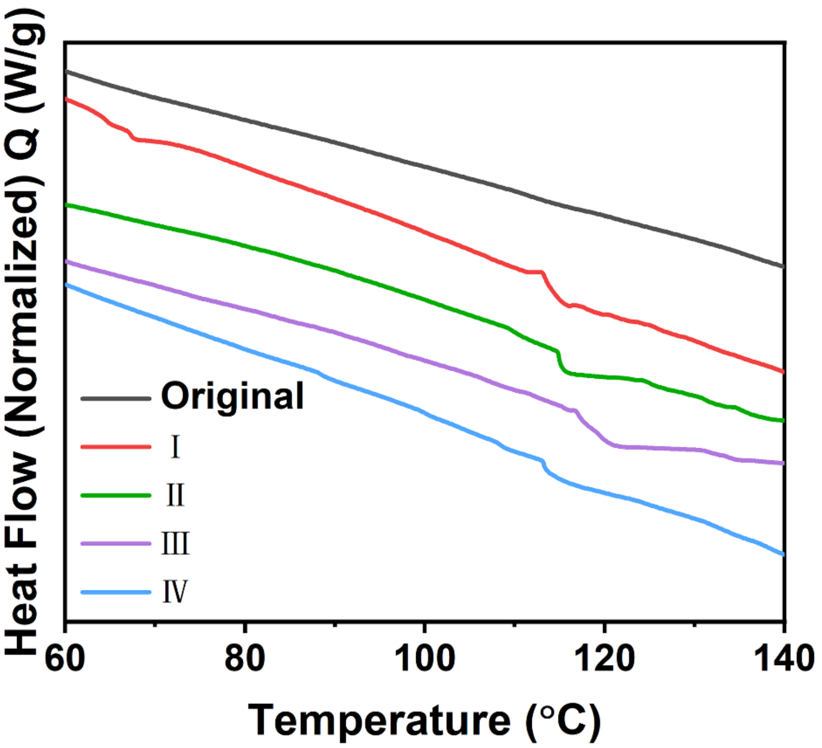

Figure S9. DSC curves of **1** at the speed of 35 ℃/min. Ⅰ, Ⅱ, Ⅲ, Ⅳ are the DSC curves of four samples after ball-drop, respectively.

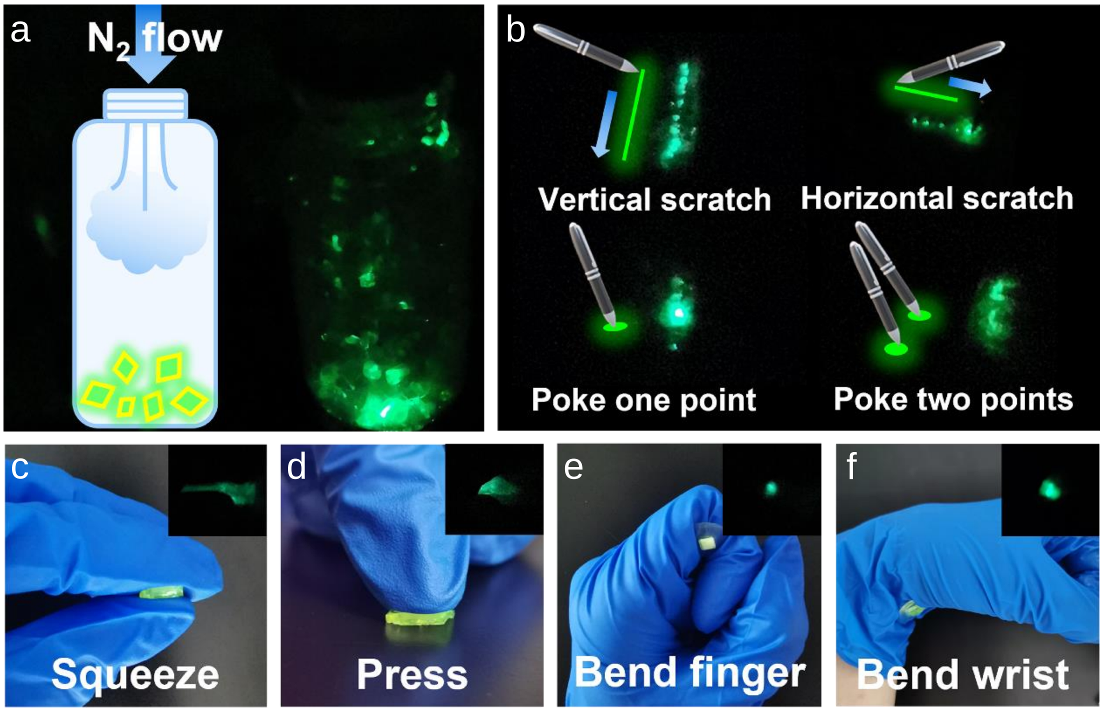

Figure S10. Visualization of mechanical stimulations. (a) The response of single crystals of polymorph **1** to nitrogen (N_2) gas flow stimulation. (b) The response of polycrystalline film to the stimulation of scratching and poking. (c-f) The response of the single crystal to the stimulation of (c) squeezing, (d) pressing, (e) finger bending, (f) and wrist bending.

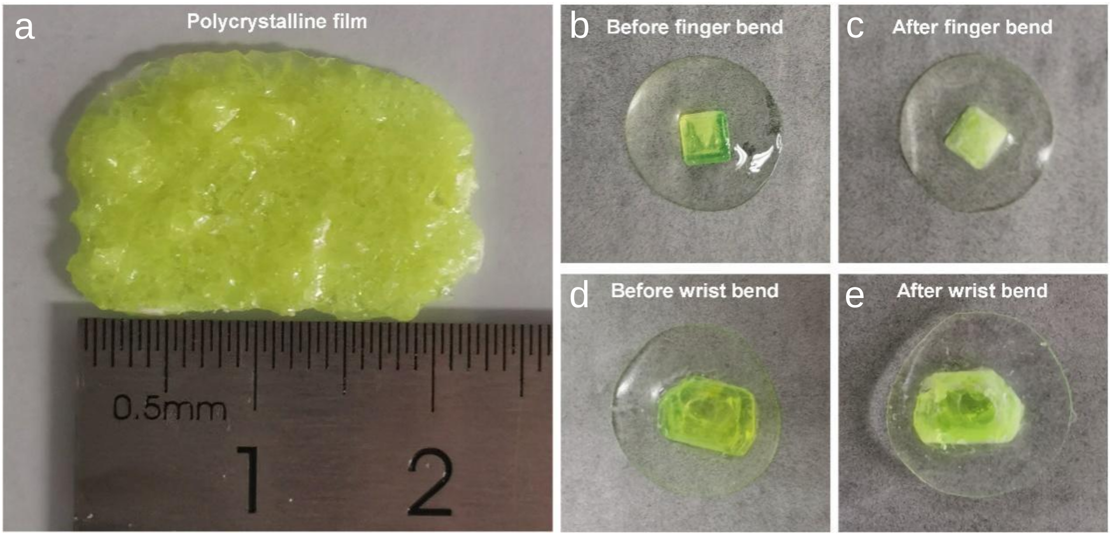

Figure S11. (a) Polycrystalline film. (b-c) The single crystal sealed with ultraviolet (UV) glue before and after finger bend, (d-e) the single crystal sealed with ultraviolet (UV) glue before and after wrist bend.

Table S1. Crystal data and structure refinement for $P2_1$ and $R\bar{3}c$.

Identification code	**1**	**2**
Empirical formula	$C_{38}H_{36}P_2MnBr_4$	$C_{38}H_{36}P_2MnBr_4$
Formula weight	929.19	929.17
Temperature/K	100.00(10)	100.00(2)
Crystal system	monoclinic	trigonal
Space group	$P2_1$	$R\bar{3}c$
a/Å	9.740(1)	10.759(5)
b/Å	12.389(1)	10.759(5)
c/Å	16.495(1)	59.276(4)
α/ °	90	90
β/ °	104.997(1)	90
γ/ °	90	120
V/Å^3	1922.54(3)	5941.9(7)
Z	2	6
ρ/g cm^{-3}	1.605	1.558
μ/mm^{-1}	8.634	4.473
F(000)	918.0	2754.0
GOF on F^2	1.062	1.145
R_1, [I > 2σ(I)][a)]	0.0276	0.0580
wR_2, [I > 2σ(I)][b)]	0.0737	0.1523

a) $R_1 = \Sigma||F_o|-|F_c||/\Sigma|F_o|$; b) $wR_2 = \{\Sigma[w(F_o^2 - F_c^2)^2]/ \Sigma[w(F_o^2)^2]\}^{1/2}$

Table S2. The specific values of d_{ij} of Crystal **1** through DFT calculations.

$$d_{ij}=\begin{pmatrix} 0 & 0 & 0 & -166.97 & 0 & -88 \\ 19.99 & 0.63 & -8.45 & 0 & 18.85 & 0 \\ 0 & 0 & 0 & -56.9 & 0 & -126.94 \end{pmatrix}$$

Table S3. Summary of elastic properties of **1**. The anisotropies are denoted by $A_X = X_{max} / X_{min}$.

Properties	Values		Directions	Anisotropies ($A_X = X_{max} / X_{min}$)
Young's modulus	E_{max}	10.02	<100>	2.67
(E, GPa)	E_{min}	3.77	<001>	
Linear compressibility	β_{max}	19.39	<001>	2.51
(β, TPa^{-1})	β_{min}	84.26	<-100>	
Shear modulus	G_{max}	39.16	(00-1) <-100>	2.85
(G, GPa)	G_{min}	1.36	(010) <-10-2>	
Poisson's ratios	v_{max}	0.70	<100, 00-1>	15.64
(v)	v_{min}	0.04	<010, -100>	

REFERENCES

1. Dolomanov OV, Bourhis LJ, Gildea RJ *et al.* OLEX2: a complete structure solution, refinement and analysis program. *J Appl Cryst*. 2009; **42**(2): 339-41.
2. Sun G, Kürti J, Rajczy P *et al.* Performance of the Vienna ab initio simulation package (VASP) in chemical applications. *J Mol Struct (Theochem)*. 2003; **624**(1): 37-45.
3. Butler KT, Svane K, Kieslich G *et al.* Microscopic origin of entropy-driven polymorphism in hybrid organic-inorganic perovskite materials. *Phys Rev B*. 2016; **94**(18): 180103.
4. Kresse G, Furthmüller J. Efficient iterative schemes for ab initio total-energy calculations using a plane-wave basis set. *Phys Rev B*. 1996; **54**(16): 11169-86.
5. Kresse G, Furthmüller J. Efficiency of ab-initio total energy calculations for metals and semiconductors using a plane-wave basis set. *Comp Mater Sci*. 1996; **6**(1): 15-50.
6. Kresse G, Hafner J. Ab initio molecular dynamics for liquid metals. *Phys Rev B*. 1993; **47**(1): 558-61.
