# Supplementary figures and images for: Self-recoverable elastico mechanoluminescence of a hybrid metal halide crystal
Source: Natl Sci Rev. 2024 Oct 21;12(5):nwae372. doi: 10.1093/nsr/nwae372 (PMC11970250; doi:10.1093/nsr/nwae372)

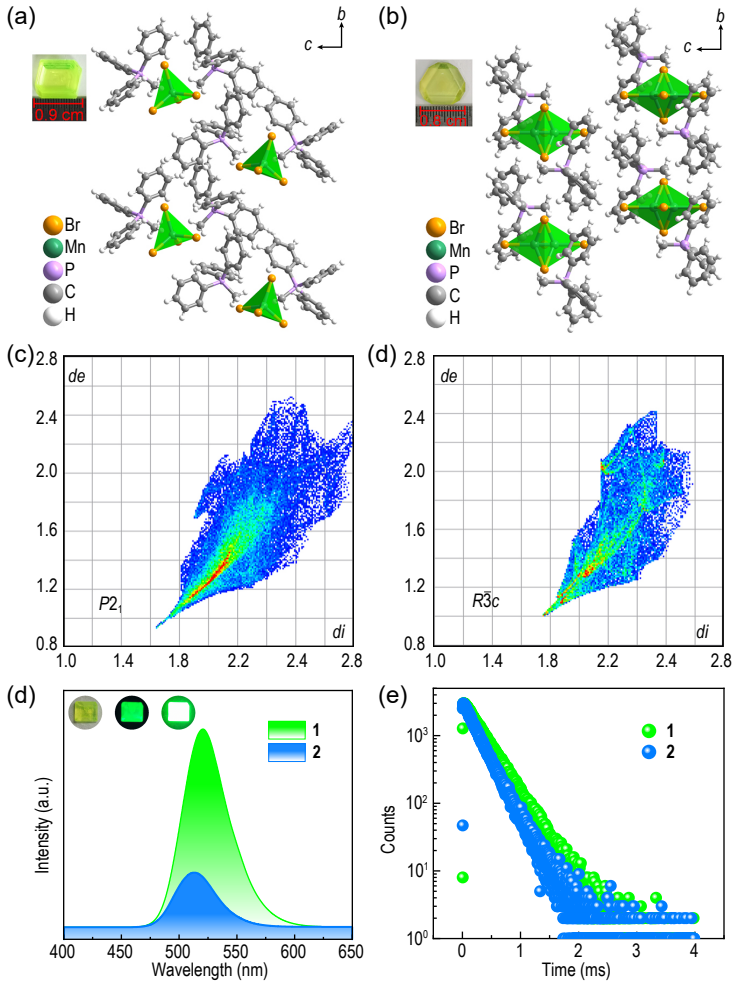

Supplement: nwae372_Supplemental_Files [file nwae372_supplemental_files.zip › fig1.margin+one column=97.5mm.2-1.pdf]
